# Rapidly Progressive Infantile Dilated Cardiomyopathy in Long–Olsen–Distelmaier Syndrome Associated with *RRAGC* NM_022157.4:c.343T>C, p.(Trp115Arg): A Case Report

**DOI:** 10.3390/genes17091106

**Published:** 2026-09-12

**Authors:** Mohammed Shahab Uddin, Fayez AlShammari, Amr Esmail, Khaled Shafeen, Fahad Alnajim, Danah AlMohaimeed, Nujud Alessa, Wojod Alothman, Fay Aldenaini, Karthiga Gurumurthy, Maryam AlQannas

**Affiliations:** 1 Department of Pediatrics, Ministry of National Guard Health Affairs, Dammam 31412, Saudi Arabia; alshammarif31@mngha.med.sa (F.A.); esmailam@mngha.med.sa (A.E.); alnajimf2@mngha.med.sa (F.A.); almuhaimeedd@mngha.med.sa (D.A.); alessan3@mngha.med.sa (N.A.); alothmanwo@mngha.med.sa (W.A.); Fay.aldenaini@gmail.com (F.A.); alqannasma@mngha.med.sa (M.A.); 2 King Abdullah International Medical Research Center, Riyadh 11481, Saudi Arabia; 3 Faculty of Medicine, King Saud bin Abdulaziz University for Health Sciences, Riyadh 11426, Saudi Arabia; 4 Faculty of Medicine, Semmelweis University, 1094 Budapest, Hungary; khaledshafeen26@gmail.com; 5 Saveetha College of Nursing, Saveetha Institute of Medical and Technical Sciences, Chennai 602105, India; gurumurthyka@mngha.med.sa; 6 Department of Nursing, Ministry of National Guard Health Affairs, Dammam 31412, Saudi Arabia

**Keywords:** cardiomyopathy, dilated, infant, newborn, genetic diseases, inborn, exome sequencing, TOR Serine–Threonine Kinases, GTP-Binding Proteins, polymicrogyria

## Abstract

**Background:** Infantile dilated cardiomyopathy (DCM) is uncommon but carries substantial mortality, particularly when accompanied by neurological, ocular, or metabolic abnormalities. Long–Olsen–Distelmaier syndrome is a rare mTORopathy caused by heterozygous gain-of-function variants in *RRAGC* and may include cortical malformations, congenital cataracts, mineral disturbances, and early-onset DCM. **Case Summary**: We describe a term male infant born to consanguineous parents with hypocalcemia and hyperphosphatemia, bilateral frontoparietal polymicrogyria, bilateral congenital lamellar cataracts, small patent ductus arteriosus and ventricular septal defect, and initially preserved biventricular function. At 53 days of age, he developed cardiogenic shock, severe lactic acidosis, and hyperkalemia during a rhinovirus/enterovirus-positive respiratory illness. Echocardiography showed severe DCM with marked left ventricular dilatation and an ejection fraction of 27%. Despite multi-agent inotropic support, ventricular function did not recover, and he died at approximately two months. Accordingly, the contemporaneous clinical WES record documented a heterozygous *RRAGC* c.343T>C, p.(Trp115Arg) variant, reported by the diagnostic laboratory as likely pathogenic. The variant was subsequently standardized in this manuscript as NM_022157.4:c.343T>C, and an independent manuscript-level ACMG/AMP reassessment was concordant. **Conclusions:** Because p.(Trp115Arg) was previously reported, the principal contribution is detailed documentation of rapid progression from preserved neonatal ventricular function to fatal infantile DCM, while extending the clinical and geographic spectrum of *RRAGC*-related Long–Olsen–Distelmaier syndrome. Respiratory viral detection should be interpreted cautiously because myocarditis was not established; early exome-based testing should be considered in infants with cardiomyopathy and multisystem abnormalities.

## 1. Introduction

Dilated cardiomyopathy (DCM) in infancy and childhood is uncommon but carries substantial mortality and transplant burden. Population-based cohorts have shown the highest incidence in infancy and death or transplantation in approximately one-third to one-half of affected children within the first several years after diagnosis [1,2,3,4,5].

Genetic evaluation is particularly important in early-onset or syndromic cardiomyopathy. Pediatric cohorts demonstrate meaningful molecular diagnostic yield, with de novo, recessive, sarcomeric, cytoskeletal, mitochondrial, metabolic, and other multisystem causes represented; moreover, evidence-based gene curation shows that only a minority of genes on broad cardiomyopathy panels have strong or definitive evidence for primary DCM [6,7,8,9,10,11,12].

Accordingly, infants with extracardiac abnormalities may benefit from broader exome- or genome-based approaches rather than narrowly restricted cardiomyopathy panels, and periodic reinterpretation can increase diagnostic yield as gene–disease knowledge evolves [13,14,15]. Current ESC guidance embeds genetic testing and counseling within cardiomyopathy care, although access to comprehensive genomic testing remains uneven across populations and health systems [16,17,18].

Within this broader landscape, a new class of “ragopathies” has emerged, linking disordered nutrient sensing to multisystem disease and cardiomyopathy. Sambri et al. (2024) reviewed monogenic disorders caused by pathogenic variants in Rag GTPases or their regulators and showed that germline gain-of-function variants in *RRAGC* and *RRAGD* can drive mTORC1 hyperactivation, TFEB inhibition, and phenotypes that include cataracts, tubulopathy, neurodevelopmental abnormalities, and dilated cardiomyopathy [19]. Long et al. (2016) first described a de novo *RRAGC* p.Ser75Tyr variant in a fetus with syndromic DCM and cataracts; mechanistic studies revealed partial amino acid insensitivity and increased mTORC1 signaling, thereby establishing gain-of-function RagC as a novel cause of syndromic pediatric heart failure [20]. Subsequently, Kim et al. (2021) demonstrated in a zebrafish knock-in model that RagC S75Y produces cardiomyopathy with impaired systolic function and reduced survival; intriguingly, mTOR inhibition with rapamycin did not rescue the phenotype, whereas TFEB overexpression restored nuclear TFEB and markedly ameliorated cardiac dysfunction, thus highlighting RagC–mTORC1–TFEB signaling as a critical therapeutic axis [21]. Likewise, Adella et al. (2024) showed that gain-of-function *RRAGD* variants induce an mTOR-dependent cardiomyopathy in zebrafish that is reversible with rapamycin, thereby reinforcing the concept that Rag-driven mTORC1 overactivation can be directly cardiotoxic [22].

Notwithstanding these advances, *RRAGC*-related disease remains exceptionally rare, and detailed pediatric cardiology-focused phenotypes have been described in only a handful of patients [19,20,21,23]. Because p.(Trp115Arg) has been reported previously, the contribution of the present case is not the identification of a novel variant; rather, it is the detailed longitudinal characterization of a neonate with congenital cataracts, cortical malformation, mineral imbalance, minor structural heart disease, and an unusually rapid transition from preserved ventricular function to fatal dilated cardiomyopathy.

## 2. Case Report

**Figure 1:**
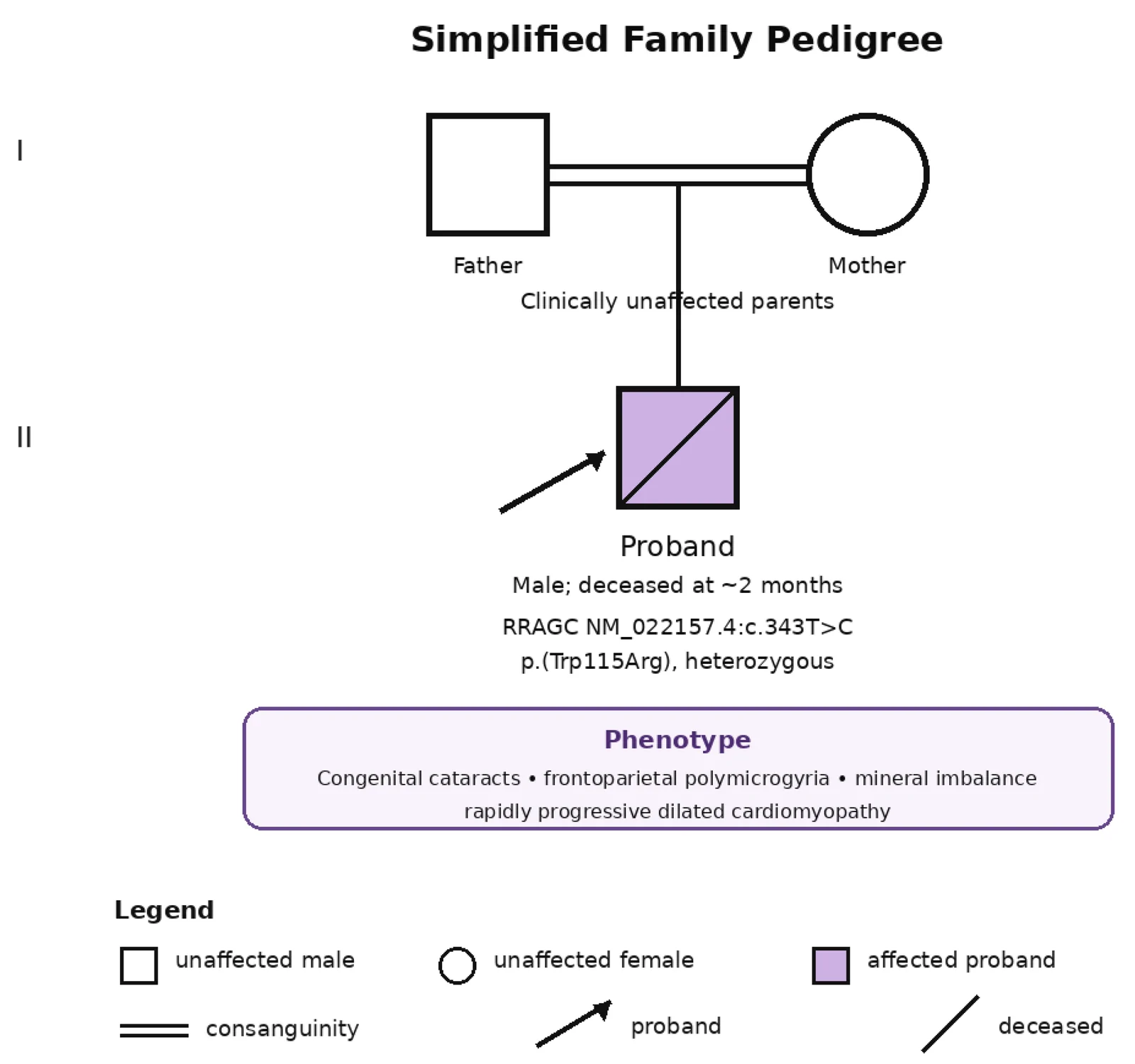
Simplified pedigree of the proband and parents. The proband was a male infant born to consanguineous, clinically unaffected parents and carried the heterozygous *RRAGC* NM_022157.4:c.343T>C, p.(Trp115Arg) variant. He presented with congenital lamellar cataracts, frontoparietal polymicrogyria, hypocalcemia with hyperphosphatemia, and rapidly progressive dilated cardiomyopathy at 53 days of age and died at approximately 2 months. The arrow identifies the proband, the diagonal line indicates death, and the double horizontal line denotes parental consanguinity. Open symbols indicate clinically unaffected parents, and the purple-filled square indicates the affected male proband. Parental segregation testing was unavailable; therefore, de novo or inherited status could not be established.

A term male infant was born to consanguineous parents (Figure 1). He required immediate admission to the Neonatal Intensive Care Unit (NICU) soon after birth, remaining there until 12 days of life. His initial postnatal course was marked by a non-blanching petechial rash and biochemical evidence of hypocalcemia with fluctuating phosphate levels, prompting endocrinological assessment and initiation of calcium supplementation. This early presentation, combined with multisystem findings, initiated a comprehensive workup to exclude congenital and structural anomalies. Initial cranial ultrasound demonstrated mildly prominent lateral and third ventricles with widened extra-axial spaces, while subsequent brain MRI, though more reassuring regarding frank cerebral atrophy, revealed bilateral frontoparietal polymicrogyria. The mineral imbalance necessitated ophthalmologic assessment, which disclosed bilateral congenital lamellar cataracts. Baseline echocardiography performed at this time showed preserved biventricular function but noted small structural heart lesions: a small patent ductus arteriosus (PDA) and a ventricular septal defect (VSD). Given this syndromic constellation—involving neurological, ocular, and cardiac findings—whole-exome sequencing (WES) was requested.

At 53 days of age, the infant presented to the emergency department with a one-day history of poor feeding, decreased activity, mild cough, and subjective fever. On arrival, he was lethargic and poorly perfused, presenting with marked tachycardia (170–190 beats/min), hypotension, and laboratory signs of lactic acidosis, raising concern for evolving cardiogenic shock. Nasopharyngeal PCR was positive for rhinovirus/enterovirus during the acute presentation. This respiratory result did not establish myocardial infection or viral myocarditis. Repeat echocardiography immediately revealed a new, severe dilated cardiomyopathy (DCM), characterized by a markedly dilated left ventricle and severely depressed left ventricular systolic function with an ejection fraction of 27%. A formal pediatric echocardiogram dated 22 May 2025 subsequently confirmed severe left-ventricular dilatation, mild left-atrial enlargement, a bicuspid aortic valve without left-ventricular outflow-tract obstruction, an unobstructed aortic arch, mild-plus tricuspid regurgitation, and no right-ventricular outflow-tract obstruction. Left-ventricular systolic function remained severely depressed, with an ejection fraction of 13% by Simpson’s method; M-mode measurements showed an ejection fraction of 26% and fractional shortening of 11%. Initial investigations confirmed a markedly elevated serum lactate of 8.7 mmol/L (reference 0.5–2.5 mmol/L), rising further despite resuscitation, and significant hyperkalemia with serum potassium levels of 7.0–7.6 mmol/L (reference 3.5–5.3 mmol/L). The deterioration occurred in temporal association with a rhinovirus/enterovirus-positive respiratory illness; however, its causal contribution and the presence of myocarditis could not be established. Cardiac biomarkers obtained during the acute presentation showed an elevated troponin I of 40 ng/L (reference 5–15 ng/L) and a markedly elevated B-type natriuretic peptide (BNP) of 1104.2 pmol/L (reference <28.9 pmol/L), indicating myocardial injury and severe cardiac hemodynamic stress but not establishing myocarditis. A contemporaneous 12-lead ECG dated 21 May 2025 showed sinus tachycardia; no ECG finding specific for myocarditis was identified. Echocardiography demonstrated global left-ventricular dilatation and severe systolic dysfunction, findings that are likewise not specific for myocarditis. No cardiac MRI, endomyocardial biopsy, myocardial viral PCR, or autopsy evidence was available. Serial echocardiographic progression is summarized in Table 1.

**Figure 2:**
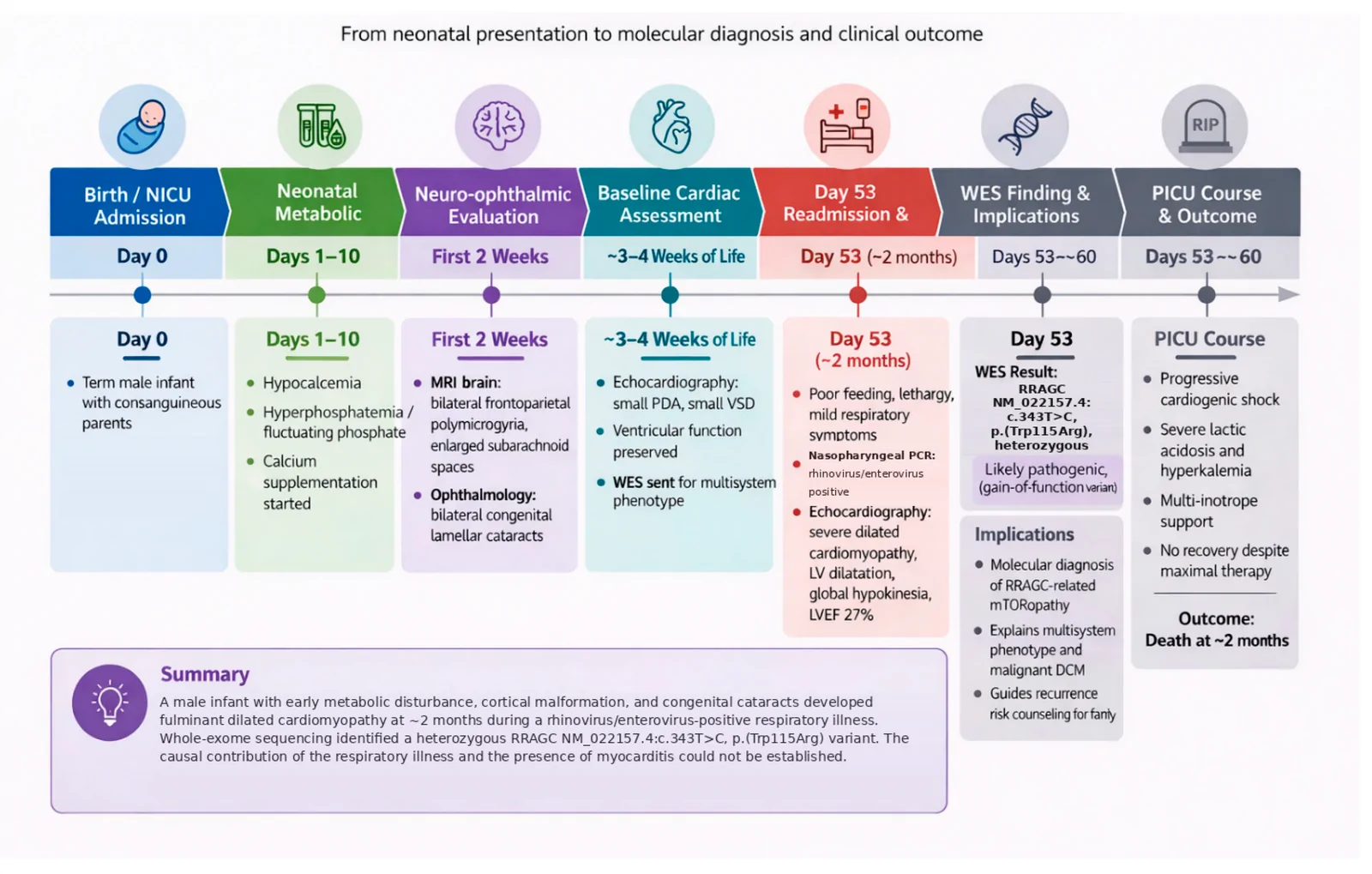
Clinical timeline of the present case. Timeline of a term male infant with early metabolic disturbance, cortical malformation, congenital cataracts, and preserved baseline ventricular function who developed fulminant dilated cardiomyopathy at 53 days of age during a rhinovirus/enterovirus-positive respiratory illness. Its causal contribution and the presence of myocarditis could not be established. Accordingly, the contemporaneous clinical WES record documented a heterozygous *RRAGC* c.343T>C, p.(Trp115Arg) variant, reported by the diagnostic laboratory as likely pathogenic and subsequently standardized in this manuscript as NM_022157.4:c.343T>C; published variant-specific evidence supports a gain-of-function mechanism consistent with the proposed unifying molecular diagnosis. Despite intensive care support, the infant developed refractory cardiogenic shock and died at approximately 2 months of age.

The infant was admitted to the Pediatric Intensive Care Unit (PICU) with a working diagnosis of acute decompensated heart failure and cardiogenic shock. Hemodynamic support was rapidly intensified, requiring multiple inotropes and vasopressors, including dopamine (8 μg/kg/min), dobutamine (4 μg/kg/min), milrinone (0.5 μg/kg/min), and epinephrine (0.1 μg/kg/min). Despite maximal medical management, serial echocardiograms showed no meaningful recovery in ventricular function, and the metabolic acidosis and hyperkalemia remained refractory to conventional measures. Given the progressive, malignant cardiac course, multiorgan involvement, and the absence of feasible curative options in this context, the multidisciplinary team and family elected to transition to comfort-focused care. The infant died in the PICU at approximately 2 months of age. The clinical timeline is illustrated in Figure 2.

### 2.1. Whole-Exome Sequencing (WES)

Methods: Clinical WES: The genomic finding originated from a pre-existing clinical whole-exome sequencing test performed on peripheral blood DNA. The available electronic clinical record documents capture-based enrichment of coding exons and flanking splice regions, paired-end short-read sequencing, alignment to GRCh37/hg19, calling and annotation of single-nucleotide variants and small insertions/deletions, filtering by sequencing quality, population frequency, predicted consequence, inheritance model, and phenotype relevance, interrogation of gnomAD, the Exome Sequencing Project, and the 1000 Genomes Project, assessment of exome-derived copy-number changes and clinically relevant coverage gaps, and interpretation under the ACMG/AMP framework.

Source verification and reporting boundary: The laboratory result was abstracted from the contemporaneous WES entry in the patient’s electronic clinical record, which was the only source available for this case report. The signed diagnostic laboratory report and raw sequencing files were not retrievable. Accordingly, we did not independently verify the primary sequencing call; locus-specific coverage, read depth, allelic balance, variant-calling metrics, assay-specific performance characteristics, and orthogonal-confirmation status were unavailable or unverified. No undocumented laboratory detail was inferred. The clinical-record result is therefore treated as secondary-source evidence and is reported separately from the fully documented manuscript-level reassessment.

Manuscript-level variant reassessment: For the independent manuscript-level reassessment, the variant was standardized as NM_022157.4:c.343T>C and NP_071440.1:p.(Trp115Arg), annotated on ENST00000373001 using Ensembl Variant Effect Predictor release 116, and queried in gnomAD v4.1.1. REVEL was used as the sole calibrated missense predictor, with evidence strength assigned according to published ClinGen-calibrated thresholds [24]. Functional evidence was evaluated using applicable ClinGen Sequence Variant Interpretation recommendations [25], whereas overall classification was synthesized using the ACMG/AMP-compatible Bayesian point framework [26]. Correlated computational predictors were not counted independently. Databases were accessed on 5 August 2026; the variant-specific evidence, criterion strengths, and point assignments are reported in the Results.

### 2.2. Results

**Figure 3:**
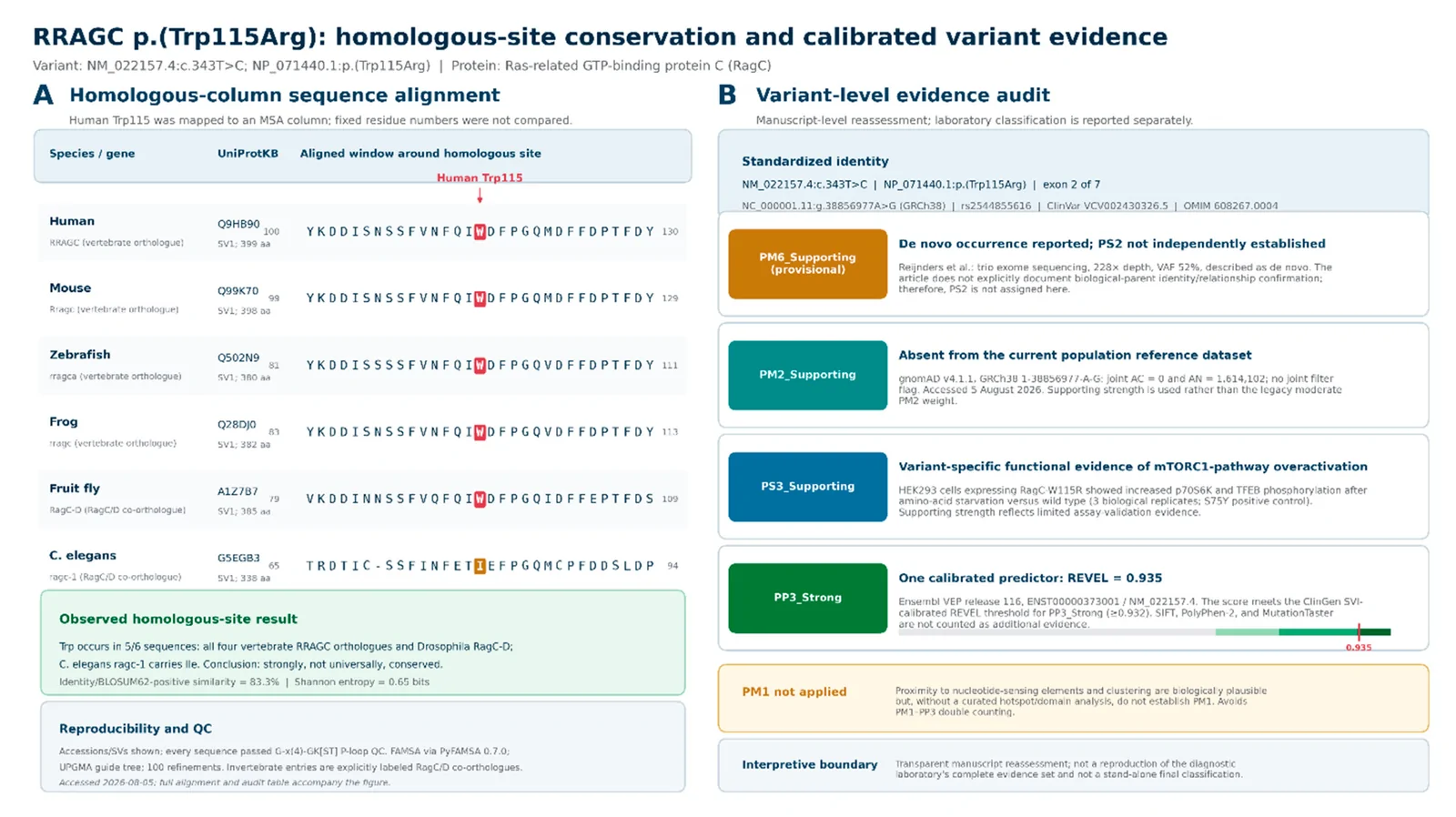
Homologous-site conservation and calibrated evidence for the *RRAGC* NM_022157.4:c.343T>C, p.(Trp115Arg) variant. (**A**) Full-length representative *RRAGC* orthologues and RagC/D co-orthologues were aligned using FAMSA. Human Trp115 was mapped to its homologous alignment column rather than compared at a fixed residue number. Tryptophan was present in five of six sequences, including all vertebrate *RRAGC* orthologues and *Drosophila* RagC-D, whereas *Caenorhabditis elegans* ragc-1 contained isoleucine; therefore, the site is strongly but not universally conserved. (**B**) The accompanying panel summarizes the variant-level evidence incorporated into the independent manuscript-level ACMG/AMP reassessment. Criterion-specific evidence, evidence strengths, supporting references, and point assignments are reported in the Results. Correlated computational predictors were not counted independently, and the panel should not be interpreted as a reconstruction of the diagnostic laboratory’s original classification.

Source of the laboratory result: The contemporaneous WES result entry in the patient’s electronic clinical record—the source document reviewed for this manuscript—documented a heterozygous missense variant in *RRAGC*, NM_022157.3:c.343T>C, p.(Trp115Arg), affecting exon 2 of 7, and recorded the diagnostic laboratory classification as “likely pathogenic” under the ACMG/AMP framework. This description reflects the verifiable content of the electronic clinical-record entry and is not presented as a direct quotation from the unavailable signed laboratory report. Therefore, no unverified laboratory name, report identifier, dates, accreditation status, assay-specific details, confirmation result, or laboratory-level criterion weighting is attributed to the diagnostic laboratory. For current nomenclature, the variant is standardized throughout the manuscript as NM_022157.4:c.343T>C, p.(Trp115Arg). The laboratory classification is retained only as a secondary clinical result and is reported separately from the independently reproducible manuscript-level reassessment described below. No additional pathogenic or likely pathogenic variants explaining the overall phenotype or providing a more plausible alternative diagnosis were documented in the available clinical-record entry. The authors independently reassessed the variant using the ACMG/AMP framework, relevant ClinGen Sequence Variant Interpretation recommendations, and the Bayesian point-based classification system. PP3_Strong was applied using REVEL as the sole calibrated missense predictor. REVEL returned a score of 0.935 for the *RRAGC* NM_022157.4:c.343T>C, p.(Trp115Arg) variant in Ensembl Variant Effect Predictor (VEP) release 116 (accessed 5 August 2026), exceeding the ClinGen-calibrated threshold of ≥0.932 for PP3_Strong described by Pejaver et al. [24]. SIFT, PolyPhen-2, and MutationTaster were not counted as additional evidence. PS3_Supporting was applied on the basis of variant-specific HEK293 experiments reported by Reijnders et al. [23], which demonstrated dysregulated mTORC1 signaling for p.(Trp115Arg); the criterion was conservatively limited to Supporting strength in accordance with ClinGen SVI functional-evidence recommendations [25]. PM2_Supporting was applied because the variant was absent from gnomAD v4.1.1 (AC = 0; AN = 1,614,102). PM6_Supporting was considered provisional because p.(Trp115Arg) had previously been reported as de novo following trio exome sequencing, but explicit confirmation of biological parental relationships was not documented. This published de novo observation pertains to a previously reported case and does not establish de novo status in the present proband, for whom parental segregation testing was unavailable. PS2, PS4, PP4, and PM1 were not applied because the available evidence did not meet the corresponding requirements. The core evidence—PP3_Strong, PS3_Supporting, and PM2_Supporting—yielded 6 points in the ACMG/AMP-compatible Bayesian point framework [26], supporting a likely pathogenic classification. Inclusion of provisional PM6_Supporting increased the score to 7 points without changing the classification. The independent manuscript-level reassessment was therefore concordant with, but methodologically separate from, the diagnostic laboratory’s original classification. The homologous-site conservation analysis and calibrated variant-level evidence are summarized in Figure 3.

*RRAGC* encodes Ras-related GTP-binding protein C (RagC), a Rag-family small GTPase that heterodimerizes with RagA or RagB and regulates amino acid-dependent recruitment and activation of mTORC1 at the lysosomal surface. Pathogenic gain-of-function variants in *RRAGC* are associated with Long–Olsen–Distelmaier syndrome, a multisystem mTORopathy characterized by cortical malformations, congenital cataracts, mineral abnormalities, and early-onset cardiomyopathy [19,20,23].

**Figure 4:**
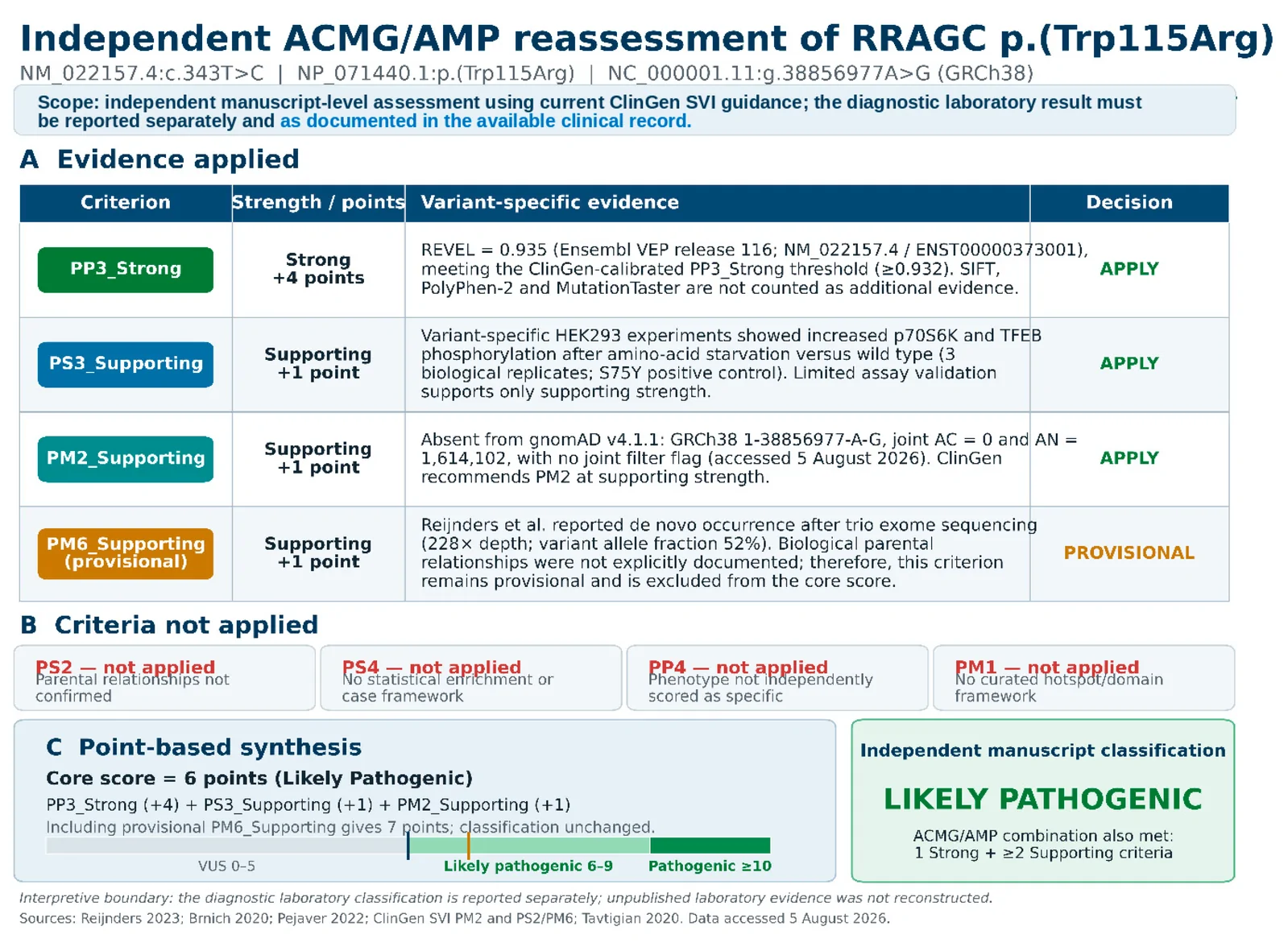
Independent manuscript-level ACMG/AMP reassessment of the *RRAGC* NM_022157.4:c.343T>C, p.(Trp115Arg) variant. (**A**) Applied evidence categories and their assigned strengths. (**B**) Criteria considered but not applied because the required evidence was unavailable or insufficient. (**C**) Bayesian point-based synthesis. The core evidence yielded 6 points, supporting a likely pathogenic classification; inclusion of provisional PM6_Supporting increased the score to 7 points without changing the classification. Detailed criterion-specific rationale and supporting references are provided in the Results. This reassessment was performed independently of the diagnostic laboratory’s original classification.

Interpretation of Genetic Findings: Overall, the independent manuscript-level reassessment was concordant with, but methodologically distinct from, the likely pathogenic classification documented in the clinical record. Accordingly, when considered together with the highly characteristic neurological, ocular, mineral, and cardiac phenotype, the molecular evidence supports *RRAGC*-related Long–Olsen–Distelmaier syndrome as the most plausible unifying diagnosis. Nevertheless, the original sequencing call was not independently verified from primary laboratory data, and inheritance remains unresolved because parental segregation testing was unavailable. Accordingly, the independent point-based classification is summarized in Figure 4.

**Figure 5:**
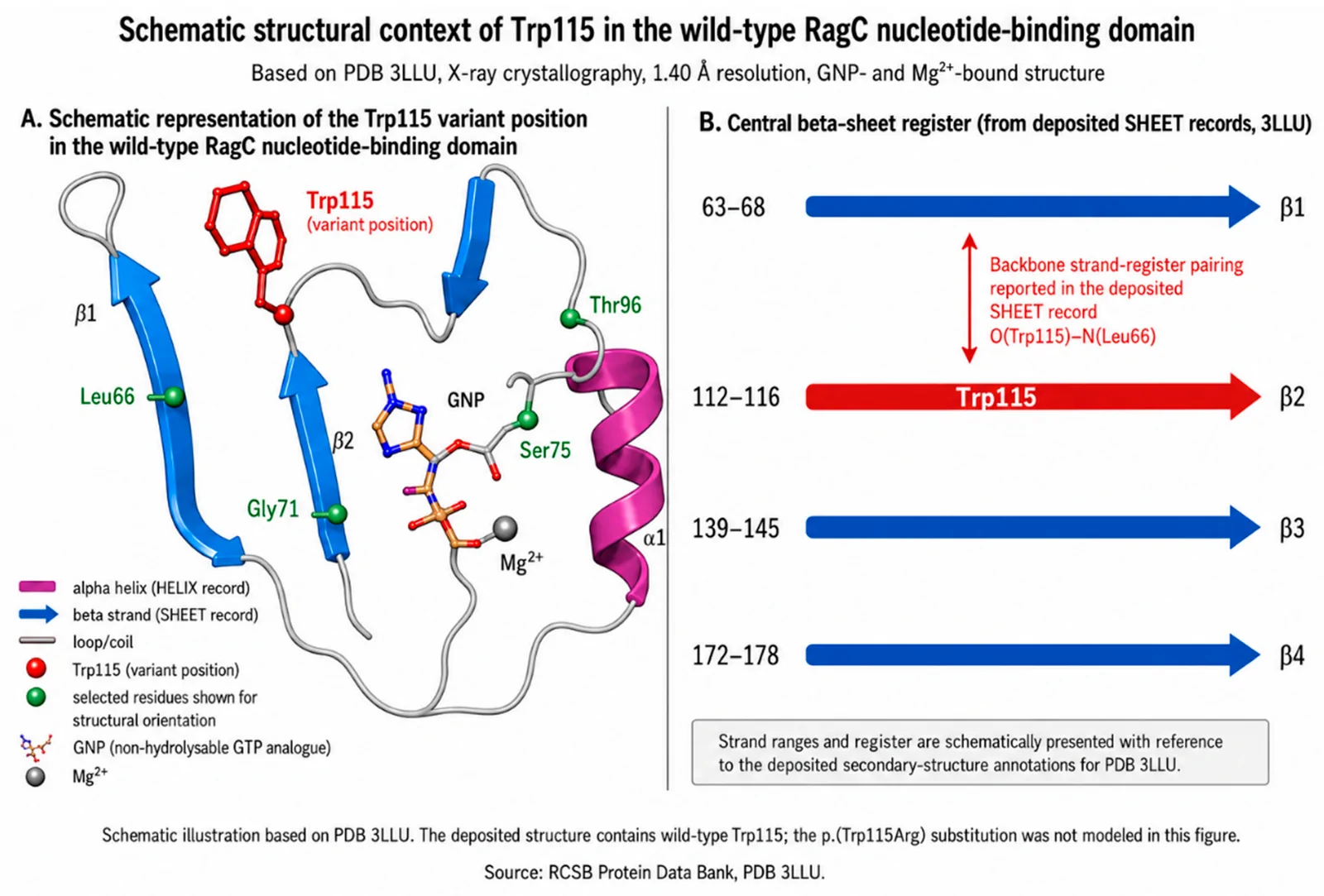
Schematic structural context of Trp115 in the wild-type RagC nucleotide-binding domain. The location of the *RRAGC* NM_022157.4:c.343T>C, p.(Trp115Arg) variant site is illustrated with reference to the experimentally determined wild-type human RagC nucleotide-binding-domain structure, PDB 3LLU, solved by X-ray crystallography at 1.40 Å resolution in the presence of GNP and Mg^2+^. (**A**) Schematic representation of the region containing Trp115. β-strands are shown in blue, α-helical structure in magenta, and connecting loops in grey. Wild-type Trp115 is highlighted in red as the position affected by the p.(Trp115Arg) substitution. GNP, Mg^2+^, and selected residues are included for structural orientation and should not be interpreted as a coordinate-derived contact analysis. (**B**) Schematic representation of the central β-sheet register with reference to the deposited secondary-structure annotations for PDB 3LLU. Trp115 is located within the strand encompassing residues 112–116. The indicated Trp115–Leu66 relationship represents the reported backbone strand-register pairing; it does not establish that the p.(Trp115Arg) substitution disrupts this interaction. The deposited structure contains wild-type Trp115, and the p.(Trp115Arg) substitution was not modelled. Accordingly, the figure is illustrative and does not constitute direct structural or functional evidence of variant pathogenicity.

To provide structural context, Trp115 was mapped onto the experimentally determined wild-type human RagC nucleotide-binding domain; however, the p.(Trp115Arg) substitution was not modeled, and the schematic does not constitute structural or functional evidence of pathogenicity (Figure 5).

Family Genetic Testing and Counseling: In view of the consanguinity, the apparent autosomal-dominant mechanism, and the severe outcome in the proband, targeted segregation analysis of the *RRAGC* NM_022157.4:c.343T>C, p.(Trp115Arg) variant in both parents was recommended to clarify inheritance (de novo vs inherited) and to refine recurrence-risk estimates. Formal results of parental testing were not available at the time of writing; consequently, inheritance of p.(Trp115Arg) in the present proband remains undetermined, and the variant cannot be designated de novo in this patient. Counseling therefore addressed the likely autosomal-dominant nature of *RRAGC*-related disease, the possibility of an unconfirmed de novo event versus inherited variation or parental germline/somatic mosaicism, and recurrence-risk uncertainty. Targeted predictive testing of at-risk relatives would be considered if segregation demonstrates an inherited familial variant; reproductive options for future pregnancies include prenatal diagnosis or preimplantation genetic testing, where appropriate.

## 3. Discussion

Infant-onset dilated cardiomyopathy (DCM) carries substantial risk, but the defining feature of the present case was the tempo of deterioration: preserved neonatal biventricular function progressed within weeks to severe DCM, refractory cardiogenic shock, and death at approximately two months of age. The accompanying cortical malformation, congenital cataracts, mineral disturbance, and cardiac disease made an underlying monogenic syndrome particularly compelling [1,2,6,7,8,9,14,27].

The p.(Trp115Arg) variant has been reported previously; therefore, this report does not introduce a novel *RRAGC* variant. Rather, the clinical and molecular findings support *RRAGC*-related Long–Olsen–Distelmaier syndrome as the most plausible unifying diagnosis and extend its natural-history evidence by documenting an unusually rapid cardiac course. Long et al. first linked gain-of-function *RRAGC* variation to syndromic fetal DCM, and Reijnders et al. later delineated p.(Trp115Arg) and related missense variants in a fatal early-onset mTORopathy; Sambri et al. subsequently placed these disorders within the broader ragopathy spectrum [19,20,23]. Our patient’s frontoparietal polymicrogyria, bilateral lamellar cataracts, mineral disturbance, congenital heart lesions, and malignant early-onset DCM are highly concordant with this reported phenotype.

From a cardiology standpoint, the tempo of ventricular deterioration in this infant is particularly striking. Initial neonatal echocardiography showed only a small patent ductus arteriosus and ventricular septal defect with preserved biventricular function; yet within a few weeks, the left ventricle became markedly dilated with an ejection fraction of 27% and no meaningful recovery despite maximal inotropic support. This rapid evolution recalls the natural history of high-risk genotypes in “classic” DCM genes such as *MYH7* or *FLNC*, in which early-onset disease can follow an aggressive course with limited reverse remodeling and a high likelihood of end-stage heart failure [11,28,29]. By contrast, many sarcomeric and cytoskeletal cardiomyopathies in older children and adults may remain relatively isolated to the heart [11,13,30]. In RagC mTORopathy, however, the cardiac phenotype is intrinsically embedded within a multisystem disorder [19,20,23]. The coexistence of cortical malformation, cataracts, mineral imbalance, and structural heart disease in our patient therefore underscores, once again, that pediatric cardiologists should maintain a high index of suspicion for syndromic mTORopathies when DCM arises in the setting of extracardiac anomalies, especially in early infancy [2,6,7,9,19,23]. The serial echocardiographic trajectory is summarized in Table 1.

The deterioration occurred in temporal association with a rhinovirus/enterovirus-positive respiratory illness; however, its causal contribution and the presence of myocarditis could not be established. A positive nasopharyngeal PCR does not demonstrate myocardial infection. During the acute presentation, troponin I was elevated at 40 ng/L (reference 5–15 ng/L) and BNP was markedly elevated at 1104.2 pmol/L (reference <28.9 pmol/L), while echocardiography documented severe global left-ventricular dilatation and systolic dysfunction. The contemporaneous ECG showed sinus tachycardia without a finding specific for myocarditis. Collectively, these findings document myocardial injury and severe hemodynamic stress but are not specific for myocarditis and cannot exclude it. No cardiac MRI, endomyocardial biopsy, myocardial viral PCR, or autopsy evidence was available. The available data did not permit characterization of the magnitude or course of the inflammatory response or virologic resolution; accordingly, no viral precipitant or “second-hit” mechanism is inferred. The syndromic phenotype and the laboratory-reported *RRAGC* result support *RRAGC*-related mTORopathy as the unifying diagnosis, while any contribution of the concurrent respiratory illness remains uncertain [19,20,23].

From a genomic standpoint, this case illustrates the value of broad sequencing when infantile cardiomyopathy is accompanied by neurological, ocular, or metabolic abnormalities. A panel restricted to canonical structural DCM genes could have missed *RRAGC*; conversely, the clinical exome-sequencing record documented a molecular finding that, when considered together with the highly concordant phenotype and the independent manuscript-level variant reassessment, provided a plausible unifying explanation for the multisystem presentation [6,7,8,13,14,23,27]. Evidence-based gene curation similarly shows that only a minority of reported DCM genes meet robust disease-association thresholds [11,12,31]. Periodic reinterpretation of genomic data may further increase diagnostic yield as gene–disease relationships and population resources evolve [15,32]; however, access to cardiomyopathy genetic testing and representative genomic research remains uneven across populations and health systems [16,17,18,33].

Figure 5 provides only positional context for wild-type Trp115 within the RagC nucleotide-binding domain. Because p.(Trp115Arg) was not modelled, the structural schematic was not used as independent pathogenicity evidence.

Experimental models implicate dysregulated RagC–mTORC1–TFEB signaling in the cardiac phenotype and provide a rationale for future pathway-directed investigation [21,22,34,35,36]. However, these approaches remain preclinical, and no therapeutic efficacy can be inferred from the present case. Additional disease-relevant cardiomyocyte and animal models will be required before genotype-directed interventions can be considered clinically [37,38].

This report has several limitations. First, it describes a single infant, and therefore the generalizability of the observations is inherently limited. Second, parental segregation testing was unavailable; inheritance of p.(Trp115Arg) remains undetermined, and the variant cannot be designated de novo in this proband. Third, the signed diagnostic laboratory report and raw sequencing files were unavailable. Accordingly, the original sequencing call, locus-specific coverage and read depth, allelic balance, variant-calling metrics, assay-specific performance characteristics, and orthogonal-confirmation status could not be independently audited; Sanger confirmation was not independently performed by the authors. The independent manuscript-level variant interpretation, by contrast, is reproducible from the reported transcript, databases, software release, evidence thresholds, criterion strengths, point assignments, and access date. Fourth, no myocardial tissue, postmortem examination, or patient-specific functional assay was available; mechanistic interpretation therefore relies on phenotype concordance and published variant-specific functional evidence rather than direct functional testing in this proband [23]. Finally, the rapidly fatal course limited longitudinal phenotyping, extended arrhythmia surveillance, and assessment of advanced mechanical circulatory support or transplantation pathways.

Future studies should prioritize additional well-phenotyped cases and disease-relevant cellular or animal models to define variant-specific mechanisms and the timing of cardiac deterioration in infancy. Such work will be necessary before pathway-directed or allele-specific interventions can be meaningfully evaluated in *RRAGC*-related disease [19,20,21,23,38].

## 4. Conclusions

In conclusion, this case supports *RRAGC*-related Long–Olsen–Distelmaier syndrome as the most plausible unifying diagnosis for a lethal multisystem phenotype that included fulminant infantile DCM. The p.(Trp115Arg) variant is not novel; the principal contribution is the documented transition from preserved neonatal ventricular function to severe, refractory DCM within the first two months of life. Concurrent respiratory viral detection remained a temporal association because myocarditis was not established. The case highlights the value of early exome-based testing in syndromic infantile cardiomyopathy and the need for further disease-specific mechanistic studies before precision therapies can be contemplated [6,7,8,14,19,20,23,27].

## Figures and Tables

**Table 1:** Serial echocardiographic progression and cardiac findings.

Time Point	Key Echocardiographic Findings	Quantitative Ventricular Function
Neonatal baseline (~3–4 weeks)	Preserved biventricular function; small patent ductus arteriosus and ventricular septal defect.	Preserved function; numerical EF not documented in the available baseline report.
Day 53 acute presentation	New severe dilated cardiomyopathy with markedly dilated left ventricle and severe left-ventricular systolic dysfunction.	EF 27%.
22 May 2025 formal pediatric echocardiogram	Severe LV dilatation; mild LA enlargement; bicuspid aortic valve without LVOT obstruction; unobstructed aortic arch; mild-plus tricuspid regurgitation; no RVOT obstruction.	LVEF 13% by Simpson’s method; M-mode EF 26%; fractional shortening 11%.
PICU serial follow-up	Persistent severe ventricular dysfunction without meaningful echocardiographic recovery.	No additional quantitative recovery documented.

## Data Availability

The data presented in this study are available within the article. Additional data are not publicly available due to patient privacy considerations.

## Figure Preparation Statement

Figure 2, Figure 3, Figure 4 and Figure 5 were generated programmatically using Python 3.x (3.12.14). ChatGPT was used to assist with development of the Python code used to construct the figures; however, the final images themselves were rendered deterministically in Python from verified source data. Matplotlib and Pillow (version 12.3.0) were used for graphical layout and rendering, pandas for organization of tabular data, and Biopython, including the Bio.PDB module where applicable, for sequence-alignment visualization and structural-data handling. All clinical values, variant annotations, sequence-alignment outputs, ACMG/AMP evidence assignments, and structural annotations were independently checked by the authors against the underlying clinical records, databases, computational outputs, and cited literature. The final figures were exported as high-resolution PNG or TIFF files for publication.

## Abbreviations

AC, allele count; ACMG/AMP, American College of Medical Genetics and Genomics/Association for Molecular Pathology; AN, allele number; BNP, B-type natriuretic peptide; DCM, dilated cardiomyopathy; ECG, electrocardiogram; ESC, European Society of Cardiology; gnomAD, Genome Aggregation Database; GTP, guanosine triphosphate; HEK293, human embryonic kidney 293 cells; ICD, implantable cardioverter–defibrillator; iPSC, induced pluripotent stem cell; LV, left ventricular; MRI, magnetic resonance imaging; mTOR, mechanistic target of rapamycin; mTORC1, mechanistic target of rapamycin complex 1; NICU, neonatal intensive care unit; PCR, polymerase chain reaction; PDA, patent ductus arteriosus; PICU, pediatric intensive care unit; REVEL, Rare Exome Variant Ensemble Learner; SIFT, Sorting Intolerant From Tolerant; TFEB, transcription factor EB; VEP, Variant Effect Predictor; VSD, ventricular septal defect; WES, whole-exome sequencing.
